# Yanyu Decoction for Aged Patients with Stable Coronary Artery Disease: A Systematic Review and Meta-Analysis

**DOI:** 10.1155/2021/6615035

**Published:** 2021-04-17

**Authors:** Shihua Shi, Zhenxing Wang, Siming Li, Xiaoping Wu, Peili Wang, Fei Wang

**Affiliations:** ^1^Hospital of Chengdu University of Traditional Chinese Medicine, Chengdu, Sichuan Province, China; ^2^National Clinical Research Center for Chinese Medicine Cardiology, Medicine Cardiology, Xiyuan Hospital, China Academy of Chinese Medical Sciences, Beijing, China

## Abstract

**Background:**

There was limited evidence of treatments aiming at aged coronary artery disease (CAD) patients. Yanyu decoction (YD) has been used as adjuvant therapy in aged patients with stable CAD and might be a new treatment worthy of recommendation for CAD patients. This study was to evaluate the combined effects of YD plus conventional pharmaceutical treatment (CPT) on senile patients with stable CAD.

**Methods:**

This review was designed according to the PRISMA (Preferred Reported Items for Systematic Reviews and Meta-Analysis) recommendations. A literature search was conducted in seven electronic databases from their inception until August 2020. Primary outcomes of interest were adverse cardiovascular events, including cardiac mortality, acute myocardial infarction (AMI), and unstable angina (UA). The secondary outcomes were blood lipids and hemorheology. Studies were pooled to calculate the risk ratio or weighted mean difference and corresponding 95% confidence interval.

**Results:**

Five studies recruiting 848 aged patients with stable CAD were included. Patients receiving YD as an adjuvant have fewer adverse cardiovascular events, including cardiac mortality, AMI, and UA. Besides, YD plus CPT has a better effect on reducing triglycerides, low-density lipoprotein cholesterol, and improving high-density lipoprotein cholesterol. Moreover, significant effects of YD plus CPT for reducing blood viscosity, plasma viscosity, and platelet aggregation rate were found compared with CPT alone.

**Conclusion:**

YD plus CPT showed better efficacy than CPT on reducing adverse cardiovascular events and improving hemorheology and blood lipids for aged patients with stable CAD. Our findings may suggest YD as an adjuvant natural-based treatment for CAD. However, more rigorous and larger trials are essential to validate our results, and further consideration of CAD studies specific to aged patients is needed.

## 1. Introduction

Coronary artery disease (CAD), recognized as a global health threat, is a chronic multifactorial disease and accounts for a high proportion of mortality in the world [1], resulting in 9.48 million deaths in 2016 [2]. Age is the strongest factor related to the development of CAD [3]. The number of people over 60 years old will surpass that of children below 5 years of age by 2020 [4]. With the aging of the global population, better strategies to treat patients of advanced age with CAD are essential. Although stable CAD is not always with an unfavorable prognosis, aged patients with stable CAD are the vulnerable population [5]. Unfavorable prognoses, including cardiac mortality, acute myocardial infarction (AMI), and unstable angina (UA), are not uncommon in aged patients with stable CAD, since aged patients with diminished physical function suffered from a combined CAD-related and age-related effect [6, 7]. Due to an increase in age, hemorheological deterioration emerges in response to aging and decline in organs or tissues [8]. Aged CAD patients usually have hyperviscosity and hyperlipemia and concentrated, aggregating, and adhesive blood, which could induce the anomaly in the vascular morphology and hemorheology in the microenvironment, thus affecting the progression and prognosis of CAD [7, 8].

Recent studies have confirmed the interaction between geriatric diseases and hyperviscosity [8] and demonstrated that blood viscosity, platelets, and hypercoagulability were related to high neutrophil levels and may increase the severity of CAD [9]. Rheological properties of blood were closely related to serum lipid levels [10]. Research has also shown that total cholesterol (TC), triglycerides (TG), and low-density lipoprotein cholesterol (LDL-C) were major factors contributing to CAD, while the high-density lipoprotein cholesterol (HDL-C) acted as a major protecting factor in CAD (Cullen et al., 1998). Aged CAD patients were identified with an anomaly in levels of TC, TG, and LDL-C. Treating aged CAD focusing on blood lipids and hemorheology is of paramount importance for public health [8].

Herbal dietary supplements continue to be accepted as an adjuvant medical option in multiple countries on CAD treatments [11]. Yanyu decoction (Yanyu Heji, YD), a formula of traditional Chinese medicine, composed of 60 grams of Stigma Maydis (*Zea mays* L.), 30 grams of Corydalis Rhizoma (*Corydalis* yanhusuo W. T. Wang), 15 grams of Fructus Trichosanthis (*Trichmanthes kiriloxvii* Maxim.), and 15 grams of Allium Macrostemon *(Allium macrostemon* Bge.), has been used in treating aged CAD patients recently. Maydis stigma, the yellowish thread from the stigmas of corn fruits, was the principal herb in YD and has been reported to contain K^+^, Ca^2+^, Mg^2+^, and Na^+^ salts, steroids, alkaloids, polyphenols, tannins, and flavonoids, with certain antioxidant abilities [12]. Some alkaloid compounds in Corydalis Rhizoma have been demonstrated to be the active constituent in the treatment of CAD and exert an antiplatelet aggregation effect [13]. Fructus Trichosanthis and Allium Macrostemon have been proven beneficial for curing CAD as a herb pair [14]. Currently, accumulating clinical evidence confirmed that YD was effective in treating stable CAD in aged patients [15–19] and revealed that YD plus conventional pharmaceutical treatment (CPT) could treat stable CAD with less adverse cardiovascular events and better effect on improving blood lipids and hemorheology.

Nevertheless, there has been no systematic review and meta-analysis available to provide evidence-based guidelines for aged CAD patients in clinical practice. Aged patients with stable CAD were underrepresented in ischemic heart disease trials because they have often been excluded from CAD trials due to higher risk, and many recommendations have been extrapolated from evidence obtained in younger cohorts [20]. Knowledge gaps in CAD have been identified, and efforts are to be paid to fill the gap.

## 2. Methods

### 2.1. Search Strategy

This review was conducted according to the PRISMA (Preferred Reported Items for Systematic Reviews and Meta-Analysis) recommendations [21]. We carried out a systematic literature search of PubMed, Cochrane Library, Web of Science, Scopus, Chongqing VIP Chinese Science and Technology Periodical Database, China National Knowledge Infrastructure Database, and Wanfang database for relevant publications from the start date of the databases until August 31, 2020. Two reviewers independently searched the studies. Any differences were resolved through discussion with another author. The following keywords were used: “Coronary Artery Disease” OR “coronary heart disease” OR “Coronary Arteriosclerosis” OR “Coronary Arterioscleroses” OR “Coronary Atherosclerosis” for CAD, “Yanyu decoction” OR “Yanyu Drug Combination” OR “Yanyu Heji” for YD, and “randomized controlled trial” OR “clinical trial” OR “RCT” for randomized clinical trials (RCTs). There were no restrictions on study time, publication language, gender, location, ethnicity, sample size, blinding methods, or treatment duration. The search results were merged and duplicate records were excluded.

### 2.2. Selection Criteria

The RCTs eligible for inclusion in the present meta-analysis had to meet the following criteria: (1) Stable CAD patients with age ≥60 years. (2) The YD group should be treated by YD plus CPT, and the control group should receive CPT the same as the YD group. (3) YD was composed of Stigma Maydis (60 g), Corydalis Rhizoma (30 g), Fructus Trichosanthis (15 g), and Allium Macrostemon (15 g). (4) YD and CPT should be given orally. (5) Primary outcomes of interest were cardiovascular events including cardiac mortality, AMI, and UA; the secondary outcomes were effective rate, blood lipids, and hemorheology. (6) No other therapies were used in the two groups.

### 2.3. Data Extraction and Quality Assessment

Two reviewers selected records and assessed research results for eligible studies independently. The information extracted from eligible studies was as follows: the first author, publication year, patient characteristics (age, gender), total number of cases, study design (interventions and duration of therapy), and reports on adverse effects. Any disagreement was resolved through discussion with another author, and final decisions were made based on consensus. We evaluated the risk of bias of the eligible trials using the Cochrane Handbook, consisting of seven domains: random sequence generation, allocation concealment, blinding of participants and personnel, blinding of outcome assessment, incomplete outcome data, selective reporting, and other bias [22]. Two researchers independently conducted the quality assessment. Another investigator was consulted if a dispute was identified.

### 2.4. Statistical Analysis

Review Manager software (Review Manager, Version 5.3, Copenhagen: The Nordic Cochrane Centre, The Cochrane Collaboration, 2014) was applied to conduct a meta-analysis, with a mean difference (MD) for continuous outcome and risk ratio (RR) for dichotomous outcomes. The size was expressed with a 95% confidence interval (CI). Heterogeneity among the studies was measured utilizing the chi-square test and the *I*^2^ statistics [23]. If significant heterogeneity was found, a random-effects model was used to compute MD and RR (*I*^2^  > 50% or *P* < 0.1). Otherwise, the fixed-effects model was applied in the absence of substantial heterogeneity [24–26].

Sensitivity analysis was carried out by omitting a single study and recalculating the pooled estimates. The results were compared with those of meta-analyses before the exclusion to figure out to what extent the excluded studies would influence the combined effect size and whether those meta-analyses were stable. Publication bias was screened if the number of included studies ≥10.

## 3. Results

### 3.1. Study Selection

**Figure 1:**
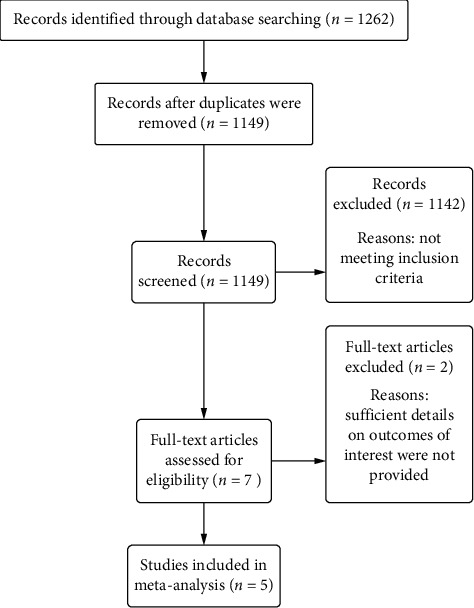
Flow diagram of search and selection process.

From a total of 1262 potentially relevant studies searched initially, 1149 records were assessed in this review after 113 duplicates were eliminated. Based on titles and abstracts, 1142 nonconforming pieces of literature were excluded for the following reasons: no control group; not elder CAD population; not YD treatment in the experiment group; combination with other drugs or any other herbs. Furthermore, 7 full-text articles were assessed for eligibility, and we excluded 2 trials because sufficient details on outcomes of interest were not provided. Therefore, a final total of 5 studies, including 424 patients in the YD group and 424 patients in the conventional drug group, were identified in the present study [15–19]. A flowchart of study inclusion is presented in Figure 1.

### 3.2. Study Characteristics

Table 1 presented the main characteristics of the included trials. All of the 5 trials were published in Chinese, with the sample size ranging from 36 to 556. The age of the total 848 participants ranged from 60 to 88 years, and the duration of treatment ranged from 28 days to 30 days. All eligible articles compared YD plus CPT with CPT alone. Four of them showed the outcomes of effective rate [16–19], and two reported the whole blood viscosity [15, 19], fibrinogen [15, 17], and platelet aggregation rate [15, 17]. Three [15, 17, 19] reported plasma viscosity, TC, TG, LDL-C, HDL-C. Four [15, 17–19] reported cardiovascular events.

### 3.3. Methodological Quality and Publication Bias

Two [17, 19] declared the generation of random sequences. None of the trials reported allocation concealment and blinding of patients, investigators, or assessors. A summary of the methodological quality assessment for each study is shown in Table 2. Publication bias was not analyzed since the number of included studies was less than 10.

### 3.4. Adverse Cardiovascular Events

#### 3.4.1. Cardiac Mortality

**Figure 2:**
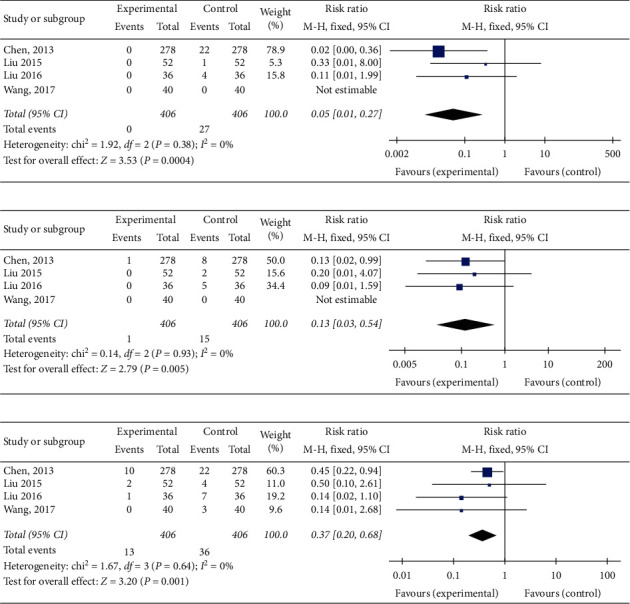
Forest plot of the comparison of Yanyu decoction versus CPT for the outcome of adverse cardiovascular events. (a) Cardiac mortality. (b) AMI. (c) UA.

Cardiac mortality was mentioned in 4 trials [15, 17–19]. The fixed-effect meta-analyses revealed that YD plus CPT may have less cardiac mortality in aged patients than CPT used alone (RR = 0.05; 95%CI: 0.01 to 0.27, *P*=0.0004; heterogeneity: *P*=0.38, *I*^2^ = 0%) (Figure 2(a)).

#### 3.4.2. AMI

AMI events were mentioned in 4 trials [15, 17–19]. Results from the meta-analyses using a fixed-effect model indicated that YD plus CPT can have fewer AMI events in aged patients than CPT used alone (RR = 0.13; 95%CI: 0.03 to 0.54, *P*=0.005; heterogeneity: *P*=0.93, *I*^2^ = 0%) (Figure 2(b)).

#### 3.4.3. UA

The cardiovascular events of UA were mentioned in 4 trials [15, 17–19]. The present study may prove YD plus CPT can have fewer UA events in patients than CPT used alone (RR = 0.37; 95%CI: 0.20 to 0.68, *P*=0.001; heterogeneity: *P*=0.64, *I*^2^ = 0%, fixed‐effect model) (Figure 2(c)).

### 3.5. Effective Rate

**Figure 3:**
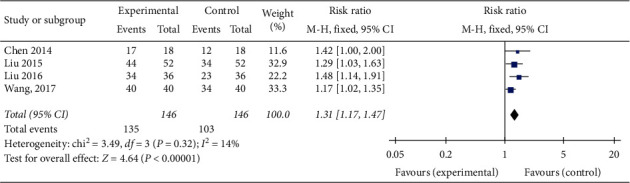
Forest plot of the comparison of Yanyu decoction versus CPT for the outcome of effective rate.

The effective rate was mentioned in 4 studies [16–19] with 292 patients. Since the absence of substantial heterogeneity (chi-square = 3.49, *P* = 0.32, *I*^2^  = 14%), a fixed-effects model was utilized for statistical analysis. Meta-analysis results revealed that compared to CPT, YD significantly improved the effective rate (RR = 1.31; 95%CI: 1.17 to 1.47, *P* < 0.00001) (Figure 3).

### 3.6. Blood Lipids

#### 3.6.1. Total Cholesterol

**Figure 4:**
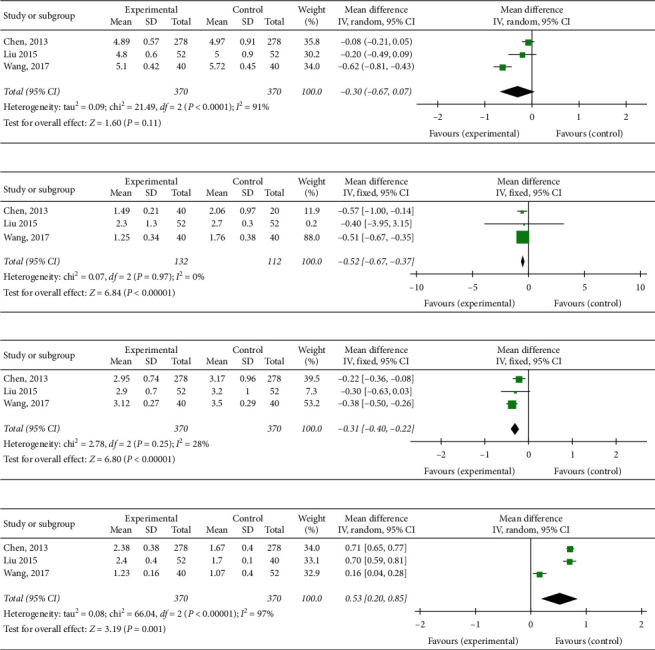
Forest plot of the comparison of Yanyu decoction versus CPT for the outcome of blood lipids. (a) Total cholesterol. (b) Triglycerides. (c) Low-density lipoprotein cholesterol. (d) High-density lipoprotein cholesterol.

Three trials [15, 17, 19] with 740 participants tested the effect of YD on TC. There were 370 patients in the YD group and control group, respectively. The analyzing result was not statistically in favor of YD plus CPT (MD = −0.30; 95%CI: −0.67 to 0.07, *P*=0.11; heterogeneity: *P* < 0.0001, *I*^2^ = 91%, random‐effect model) (Figure 4(a)).

#### 3.6.2. Triglycerides

There were 3 studies [15, 17, 19] that provided relevant data for triglycerides. There were 132 patients in the YD group and 112 patients in the CPT group. Compared with CPT used alone, a noteworthy lowering effect on TG in favor of YD therapy was observed after the treatment (MD = −0.52; 95% CI: −0.67 to −0.37; *P* < 0.00001; heterogeneity: *P* = 0.97, *I*^2^  = 0%, fixed‐effect model) (Figure 4(b)).

#### 3.6.3. Low‐Density Lipoprotein Cholesterol

There were 3 studies [15, 17, 19] evaluating the effectiveness of YD on LDL-C when compared with CPT alone. There were 370 patients in the YD group and 370 patients in the pharmaceutical group. Noteworthy lowering on LDL-C in favor of YD therapy was observed after treatments (MD = −0.31; 95% CI: −0.40 to −0.22; *P* < 0.00001; heterogeneity: *P* = 0.25, *I*^2^  = 28%, fixed‐effect model) (Figure 4(c)).

#### 3.6.4. High‐Density Lipoprotein Cholesterol

Three trials [15, 17, 19] with 740 patients reported HDL-C. There were 370 patients treated by YD plus CPT and 370 patients treated by CPT. According to the test of heterogeneity (chi-square = 66.04, *P* < 0.00001, *I*^2^  = 97%), a random-effects model was utilized to accommodate heterogeneity. The analyzing result was statistically in favor of YD plus CPT (MD = 0.53; 95%CI: 0.20 to 0.85, *P* = 0.001) (Figure 4(d)).

### 3.7. Hemorheology

#### 3.7.1. Whole Blood Viscosity

**Figure 5:**
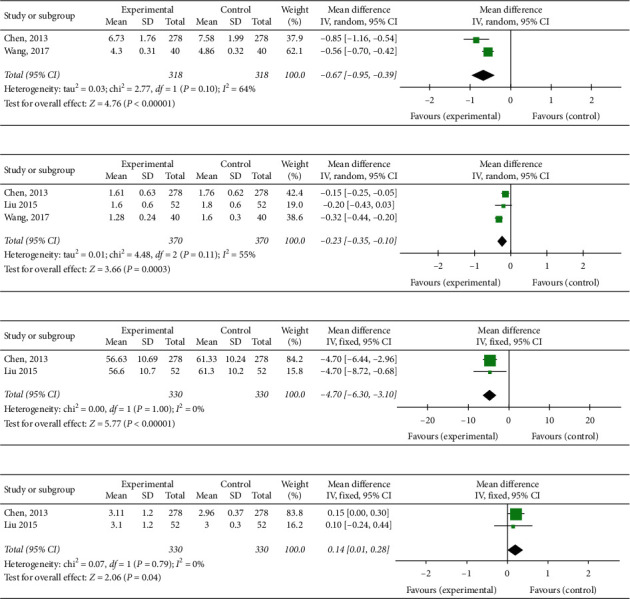
Forest plot of the comparison of Yanyu decoction versus CPT for the outcome of hemorheology. (a) Whole blood viscosity. (b) Plasma viscosity. (c) Platelet aggregation rate. (d) Fibrinogen.

Two trials [15, 19] with 636 patients reported blood viscosity. There were 318 patients treated by YD plus CPT and 318 patients treated by conventional pharmaceutical therapy. According to the test of heterogeneity (chi-square = 2.77, *P* = 0.10, *I*^2^ = 64%), a random-effects model was utilized for statistical analysis. The combined effects of these two independent trials might show a significant lowering effect of YD plus CPT on whole blood viscosity in stable CAD patients when compared with CPT alone (MD = −0.67; 95%CI: −0.95 to −0.39, *P* < 0.00001) (Figure 5(a)).

#### 3.7.2. Plasma Viscosity

The effectiveness of YD on plasma viscosity rate was evaluated in 3 trials [15, 17, 19]. There were 370 patients in the YD groups and 370 patients in the control groups, respectively. A random-effects model was used for statistical analysis based on the test of heterogeneity (chi-square = 4.48, *P*=0.11, *I*^2^  = 55%). The combined effects of these 3 independent trials suggested lowering effects of YD plus CPT on plasma viscosity in patients when compared with CPT alone (MD = −0.23; 95% CI: −0.35 to −0.10, *P*=0.0003) (Figure 5(b)).

#### 3.7.3. Platelet Aggregation Rate

Two studies [15, 17] evaluating the effectiveness of YD on platelet aggregation rate when compared with CPT alone. A sum of 330 patients in the YD group and 330 patients in the pharmaceutical group. A noteworthy lowering effect on platelet aggregation rate in favor of YD therapy was observed after the treatment (MD = −4.70; 95% CI: −6.30 to −3.10; *P* < 0.00001; heterogeneity: *P*=1.00, *I*^2^  = 0%, fixed‐effect model) (Figure 5(c)).

#### 3.7.4. Fibrinogen

Two trials [15, 17] with 660 participants tested the effect of YD on the fibrinogen. There were 330 patients in the YD group and control group, respectively. The combined effects might show a significant improving effect of YD plus CPT on fibrinogen in stable CAD patients when compared with CPT alone (MD = 0.14; 95%CI: 0.01 to 0.28, *P*=0.04; heterogeneity: *P*=0.79, *I*^2^ = 0%, fixed‐effect model) (Figure 5(d)).

### 3.8. Generic Adverse Events

Only two articles [15, 16] reported generic adverse events, of which one [15] reported that nausea, dizziness, or palpitations occurred in the YD group but disappeared without any treatment in a short time. However, the other [16] reported that no adverse events occurred in both the YD group and the control group. Whether YD plus CPT has fewer generic adverse events than CPT used alone or not cannot be evaluated.

### 3.9. Sensitivity Analyses

Sensitivity analysis was conducted to evaluate the robustness of merged results. There was little difference in adverse cardiovascular events, effective rate, and hemorheology between original meta-analyses and reevaluation. The sensitivity of the results about adverse cardiovascular events, effective rate, and hemorheology was relatively low, and the results were stable. However, with regard to blood lipids, the combined effect size was changed when the possible anomalous studies of TC [17, 19] and HDL-C [15, 17] were removed, indicating that more information about TC and HDL-C was required.

## 4. Discussion

### 4.1. Interpretations

CAD is a major cause of morbidity and mortality in aged patients [27]. Aging is an inevitable part of life, with a remarkable effect on the heart and arterial system, leading to an increase in cardiovascular diseases like atherosclerosis and myocardial infarction [28]. Nonetheless, numerous CAD studies have excluded aged patients, leading to uncertainty about the efficacy and safety of clinical treatments [29]. As aged patients with functional declines are the fastest growing population dying of CAD [30], further improvements in prevention, diagnosis, and treatment of CAD among the aged are needed to address these gaps.

Dyslipidemia is an established risk factor for CAD that is frequent in patients of advanced age [31]. The pathogenesis of CAD in aged patients is closely correlated with the level of blood lipids and hemorheological indicators. Pathways involving lipid metabolism and coagulation play important roles in vascular aging [32]. The adenosine monophosphate-activated protein kinase (AMPK), involved in lipid metabolism, is cardioprotective during ischemia and reperfusion [33]. Mouse models using dominant-negative AMPK exacerbated myocardial infarction [34]. Modifying lipid profiles can reduce coronary morbidity and mortality [35] and more aggressive therapeutics on lowering LDL-C are emerging, e.g., PCSK9 inhibitors [36]. However, high cost and repeated injection reduced its availability for aged patients. YD, a plant-based regimen, is less costly and shows the beneficial effect for aged patients with CAD. The principal herb of YD is Stigma Maydis, which is also an easily available food with good taste. Stigma Maydis plays a critical role and the dosage of it is the largest in YD. Aged patients are at higher risk for multiple diseases at the same time. In addition to alleviating CAD, Stigma Maydis has a therapeutic effect on hypertension, edema, diabetes, and gastritis, which usually occur in aged CAD patients [12, 37]. Based on traditional Chinese medicine (TCM) theory, Corydalis Rhizoma has been commonly used in clinic for the treatment of angina with the function of activating blood circulation to dissipate blood stasis and relieve pain. According to TCM, the pathological feature of CAD was much phlegm and stasis, which resulted in chest pain. YD, especially suitable for CAD patients with the syndrome of qi stagnation, blood stasis, and phlegm obstruction, could regulate the flow of qi, reduce dampness, and dispel pathogenic factors. However, lack of relevant animal experiments and in vitro cell tests, the pharmacological mechanism of YD has not been investigated, and the potential molecular intervention mechanisms are unclear. Our study may inspire future researches in this direction.

Although YD has been applied in stable CAD treatments in recent years, there was not any systematic review and meta-analysis to summarize the clinical effects of YD on stable CAD. To the best of our knowledge, the present study is the first of its kind to provide an evidence-based approach to the treatments of aged patients with CAD and may suggest YD as a new adjuvant herb treatment for aged patients with stable CAD.

Unfortunately, the heterogeneity of TC and HDL-C was high. We found that the heterogeneity of TC and HDL-C was reduced to 0 when one study [19] was excluded. But when we compared this study with other pooled studies, no significant clinical heterogeneity was found, considering all included studies have the same prescription of Chinese herbal medicine and disease, and the patients and treatment duration were similar among them. Although we used a random-effects model to accommodate heterogeneity, the sources of heterogeneity were not found. Thus, the certainty of evidence relevant to TC and HDL-C may be degraded.

### 4.2. Strengths and Limitations

There were several strengths related to our study. Firstly, this was the first study systematically reviewing YD in aged stable CAD patients. We used a broad range of search terms and conducted a comprehensive systematic search, including a variety of databases. We founded that only a small number of clinical studies explored pharmaceutical treatments for aged CAD patients, demonstrating the existence of an important gap in the literature. For aged CAD patients, the guidelines providing precise treatment recommendations were not available, and relevant recommendations for treatments can only be based on previous literature. This systematic review and meta-analysis described and evaluated the available clinical trials, providing a comprehensive synthesis of up-to-date evidence of CAD trials focusing on aged patients to fill the gap in the literature. Secondly, independent investigators performed the study selection and quality assessment and demonstrated YD as adjuvant therapy in treating stable CAD among aged patients. YD plus CPT may reduce adverse cardiovascular events, which provided aged CAD patients a new choice in treatments. YD plus CPT might have a better effect on reducing TG, LDL-C and improving HDL-C and hemorheology. YD might address the disorder in hemorheology and dyslipidemia, the key contributors to atherosclerosis in aged patients, which has far-reaching effects. Aged CAD patients may have fewer adverse cardiovascular events using YD as an adjuvant medicine. The improvements in blood lipids and improved blood flow brought by YD may be promising and impressing in CAD treatments. While current studies are still limited, it is expected that the importance of YD for aged patients with CAD will be increasingly appreciated in the coming years. Our research may provide a new direction and focus in future CAD studies.

Some limitations should be considered in our study results. As demonstrated in this review, only five dedicated studies evaluating the treatment of CAD in aged patients were reported, though researches regarding this issue are extremely important. No RCTs about YD in another country out of China have been found up to now. Only five Chinese studies were analyzed, which may induce the potential bias of the study. Whether YD can be used in more ethnic groups needs further studies. In addition, whether YD plus CPT has fewer adverse events or not is still not unequivocal. Moreover, positive results tend to be reported more frequently and the efficacy of YD might be overestimated. Furthermore, it is hard to conduct double-blind clinical trials studying herb decoction. The methodological quality is poor according to the Cochrane Collaboration's tool.

## 5. Conclusion

In summary, YD plus CPT may have a better effect on reducing cardiac mortality, AMI, and UA in elder stable CAD patients when compared with CPT alone. Besides, it might also be helpful for not only reducing TG, LDL-C and improving HDL-C but lowering blood viscosity, plasma viscosity, and platelet aggregation rate. YD may be beneficial for stable CAD, though some limitations might weaken the validity of these findings. More RCTs of high-quality and more carefully designed clinical trials are recommended to generate a high level of clinical evidence to confirm these findings.

## Figures and Tables

**Table 1:** Characteristics of the included trials.

References	Number of subjects		Intervention		Age: Mean ± SD		Male/Female		Treatment duration
	E	C	E	C	E	C	E	C	
Chen et al. [15]	278	278	YD	CPT	69^†^ (60–82)*∗*	72^†^ (61–88)*∗*	171/107	163/115	30d
Chen [16]	18	18	YD	CPT	68.54 ± 4.14 (62–78)	68.98 ± 4.76 (61–79)	10/8	11/7	30d
Liu [5]	52	52	YD	CPT	67.5 ± 11.3 (62–78)	68.5 ± 12.6 (60–79)	33/19	35/17	30d
Liu [18]	36	36	YD	CPT	66.5 ± 1.2 (61–77)	67.9 ± 2.5 (60–78)	22/14	20/16	1M
Wang and Yang [19]	40	40	YD	CPT	68.0 ± 4.0 (61–79)	68.2 ± 6.5 (62–77)	22/18	23/17	28d

*C* = control group, YD = Yanyu decoction, *d* = days, *E* = experiment group, *M* = month, NA = not applicable, CPT = conventional pharmaceutical treatment, SD = standard deviation, *y* = years, ^†^Median, *∗*range. YD: Stigma Maydis 60 g, Corydalis Rhizoma 30 g, Fructus Trichosanthis 15 g and Allium Macrostemon 15 g, 1 dose/day, bid.

**Table 2:** Methodologic quality of the included trials based on the Cochrane handbook.

References	A	B	C	D	E	F	G
Chen et al. [15]	−	?	?	?	+	+	+
Chen [16]	?	?	?	?	+	+	+
Liu [5]	+	?	?	?	+	+	+
Liu [18]	−	?	?	?	+	+	+
Wang and Yang [19]	+	?	?	?	+	+	+

*A* = Random sequence generation (selection bias); *B* = Allocation concealment (selection bias); *C* = Blinding of participants and personnel (performance bias); *D* = Blinding of outcome assessment (detection bias); *E* = Incomplete outcome data (attrition bias); *F* = Selective reporting (reporting bias); *G* = Other bias; +, low risk; −, high risk; ?, unclear.
